# Triage of urology service to cope with COVID-19 pandemic: A single institution study

**DOI:** 10.17179/excli2020-3135

**Published:** 2021-01-07

**Authors:** Muhammad Waqar, Kelly Ong, Amr Moubasher, Omer Farooq Rehman, Kamran Faisal Bhopal, Jonathan Makanjuola

**Affiliations:** 1 Department of Urology, King's College Hospital, London, UK; 2 Armed Forces Institute of Urology, Rawalpindi, PAK; 3 Addenbrooke's Hospital, Cambridge University Hospitals NHS Foundation Trust Cambridge, UK

**Keywords:** COVID-19, bladder cancer, kidney cancer, testicular cancer

## Abstract

Almost a year ago, no one has ever heard of COVID-19 but now, every individual in the world is familiar with this term. It is far from over and yet, it has affected every aspect of human life. The Department of Urology at King's College Hospital London provides all types of urology care ranging from benign to cancer treatments to the community. However, this service was badly affected by COVID-19. Policies were made by the experts in the field to reduce patient traffic in the hospital and at the same time, attempting to ensure appropriate and timely treatment was provided to patients suffering from urological conditions requiring urgent attention. In this article, we discuss the triage guidelines set up at our centre. Treatments for benign conditions such as kidney stones were delayed for 3-6 months. For the first time, telephone and video clinics were setup to follow-up patients with benign conditions. Urological emergencies such as acute urinary retention and priapism were discharged from accidental and emergency department after treatment. Small T1 renal cancers were put on surveillance, whereas T2 and T3 renal cancers were offered nephrectomy at a COVID-free specialized center. Transurethral removal of bladder tumor was offered only for solid or actively bleeding tumor. High risk prostate cancer patients were started on hormonal therapy and radiotherapy was only offered for spinal cord compression secondary to metastasis. Low and intermediate non-metastatic prostate cancers were placed on active surveillance. Patients with testicular tumor continued to have immediate inguinal orchidectomy. The multi-disciplinary meetings were done remotely using blue jeans software®. These steps not only strive to provide adequate and timely urology care to patients but also protect health care workers and prevent the spread of COVID-19.

## Introduction

The COVID-19 pandemic caused thousands of deaths worldwide to date and the death toll continues to rise. Health systems across the world felt tremendous pressure in dealing with this crisis. The pathogen, first identified by China, was declared as a pandemic in March 2020 by the World Health Organization (WHO). Very rapidly, the health care system globally has become overwhelmed. Hospitals were not only treating COVID-19 patients but also became a dangerous place for transmissions. Therefore, it is very important to keep people away from hospital as much as possible, in the safest possible way. All medial specialty services were affected by it. We evaluated the effect of COVID-19 on our local urology service in King's College Hospital London. Local guidelines were created based on expert opinions to provide protection to our health care system without compromising the safety of our patients. In this article, we are going to discuss these urology triage guidelines and their possible outcome.

## Materials and Methods

### Benign conditions

#### Stone

We observed an overall decrease in trend of patients presenting with renal colic via accident and emergency. We speculated that it is secondary to patients' avoidance of hospital visit for fear of contracting COVID-19. Ureteric stone patients without sepsis or acute kidney injury (AKI) were mainly managed conservatively and were only offered intervention if they had failed conservative treatment. For septic or obstructive uropathy secondary to stone, nephrostomy was the preferred intervention. If nephrostomy could not be performed, then primary ureteroscopy was favored over stenting. This was to avoid repeated general anesthesia in order to prevent risk of aerosolization and subsequent cross contamination. For radiopaque ureteric stone, patients were referred for extracorporal shockwave lithotomy (ESWL) at a different specialist hospital (Guy's and St Thomas' NHS Foundation Trust).

#### Acute urinary retention

Acute urinary retention patients were discharged directly from accident and emergency department after ensuring they were not diuresing. To safeguard patients from the risk of developing AKI, these patients were brought back to surgical assessment unit for repeat blood test the next day. Hospital admission was avoided where ever possible.

### Other emergencies

#### Priapism and testicular torsion

Priapism was not a clinical presentation which commonly required admission even in pre-pandemic times. Therefore, the management of this condition remained largely unaffected by the pandemic. After needle aspiration, they were all discharged from the accident and emergency department.

#### Malignant conditions

According to a Chinese study, cancer patients were more prone to acquiring COVID-19 due to their suppressed immunity from their disease and secondly due to the treatments they received such as chemotherapy, radiotherapy and surgery. A study showed that cancer patients with COVID-19 had 3.5 times higher chances of requiring mechanical ventilator or ITU admission or dying compared to non-cancerous patients (Liang et al., 2020[9]). Urological cancers form the main bulk of urological surgeries during the pandemic. However, around 68 % of these surgeries can be safely postponed as shown in a recent Italian study (Campi et al., 2020[5]). Expert opinions and the British Association of Urological Surgeons (BAUS) COVID-19 forum were used to create guidelines to keep urological cancer services running to ensure patient safety.

#### Kidney cancer

A study published in 2016 shed light on the effects of delaying surgery in renal cancer patients. Mano et al study concluded that renal cell cancers of less than 4 cm had no effect on outcome if it was delayed, however, survival rate in tumors bigger than 4 cm were negatively affected if there was a delay in their surgical times (Mano et al., 2016[10]). Imaging for suspected T1a-T1b renal tumors and complex cysts were delayed for 6-9 months. Confirmed cases were placed on surveillance. T2 and T3 renal cancer patients were offered nephrectomy. Newly diagnosed metastatic renal cancer patients were started on systemic therapy. Follow-up scans for low and intermediate risk renal cancer patients were postponed for 6 months due to the decreased scanning resources. Imaging for follow-up of high risk renal cancer patients were deferred for 3 months. These policies could increase anxiety in patients with small renal cancer due to the unpredictability of the outcome although the chances of metastasis is less than 1 % (BAUS, 2020[3]).

#### Bladder cancer

Bladder cancers are mostly non muscle invasive bladder cancer (NMIBC) and are found more commonly in men than women. 63 % of bladder cancer patients have at least a single comorbidity, whereas 32 % of them have two or more comorbidities (Goossens-Laan et al., 2014[6]). There is 15-40 % chances of NMIBC to progress to muscle invasive disease and approximately 20 % of these people die from bladder cancer (Klaassen et al., 2018[8]; Thomas et al., 2013[13]). Keeping this in mind, hematuria clinics were not cancelled as it was felt that identification and treatment of potential bladder cancers were important and time critical. Most common presenting symptom for bladder cancer is painless hematuria. Hematuria clinics are the screening platform for bladder cancer. Due to the pandemic, these clinics were limited to investigate visible hematuria only. Non-visible hematuria investigations were deferred for 6 months. Transurethral removal of bladder tumor (TURBT) were offered only for solid or actively bleeding tumor. Further treatment was guided depending on histology, comorbidities and age of patient.

For low and intermediate NMIBC, their first flexible cystoscopy was delayed for 6-12 months. The consequences of this delay in investigation such as not picking up early recurrences are debatable. For high risk bladder cancer, flexible cystoscopy has been planned after 3 months (BAUS, 2020[2]).

Ideal treatment for MIBC is either bacillus Calmette-Guérin (BCG) or radical cystectomy (Babjuk et al., 2019[1]). BCG therapy is an immunotherapy which is seen most effective during their induction and 1^st^ maintenance dose (Kamat et al., 2017[7]). Due to this fact, we discontinued BCG therapy for all the patients who had received induction and 1^st^ dose of their mountainous therapy. For high risk muscle invasive bladder cancer patients, treatment with BCG induction and 1^st ^dose continued. Resection was deferred for a few months. High risk bladder cancer patients with little comorbidities were offered radical cystectomy. Metastatic bladder cancer patients were offered chemotherapy.

#### Prostate cancer

BAUS new guidelines were followed for managing high prostate specific antigen (PSA) level referrals. Patients with PSA less than 20 had complete urological assessment including trans-rectal ultrasound of prostate to calculate PSA density. If it was > 0.15, trans-perineal biopsies were arranged. No biopsy was offered in men with significant comorbidities. TRUS biopsies were completely avoided during this period. Patient with PSA density of < 0.15 were reassured and discharged with the advice of getting their PSA rechecked in community in 6 months. Patients with PSA greater than 20 were arranged to have a bone scan to assess metastasis and started on hormone therapy with a repeat PSA in 3-6 months. Chemotherapy was avoided due to immunosuppressive risk. Radiotherapy was only offered for spinal cord compression secondary to metastasis. Low and intermediate risk of non-metastatic prostate cancers were placed on active surveillance. High risk patients were started on hormonal therapy until radical prostatectomy or radiotherapy could be offered safely. Follow-up appointments were deferred for 3-6 months (BAUS, 2020[4]).

#### Testicular cancer

About 9500 men in the United States were diagnosed with testicular cancer in 2018. The key type of testicular cancer is testicular germ cell tumor. Seminomas and nonseminomas are the two major types of testicular germ cell tumors, with an incidence of 13 % and 55 %, respectively.Treatment starts with inguinal orchidectomy for all testicular germ cell tumors (Miller et al., 2019[11]). There are limited studies with regards to the outcome of delayed intervention in testicular cancer. Inguinal orchiectomy was carried out once testicular cancer diagnosis was confirmed. This was because most of testicular cancer patients are young and delaying it could have fatal outcomes. Pure seminomas were placed on active surveillance rather than adjuvant carboplatin chemotherapy. Non-seminoma germ cell tumors were also moved to active surveillance but for non-compliant patients, adjuvant single-cycle chemotherapy (BEP) was given along with self-isolation. No changes were made for metastatic testicular tumor. They were all either given chemotherapy or radiotherapy (Ward, 2020[14]). We have also observed increase in trend of patients presenting with non-specific testicular pain. It is not possible to exclude this as another unreported symptom of COVID-19 as these patients were not tested due to lack of testing kits. These guidelines were based on expert opinion and BAUS guidelines.

## Results

Long-term outcomes of these measures are unknown. COVID-19 has mostly negative impact on our services; nonetheless, we discovered some novel practices which were effective. Multi-disciplinary meetings were done remotely using blue jeans software®. This enabled the team to attend meetings from home which was time-conserving. This was popular amongst our urology colleagues and has been recommended to adapt this practice permanently as it not only limits physical interaction but also a very efficient way of communication.

### Changes in operating frame work

Patients booked for surgery were asked to self-isolate for 2 weeks prior to surgery. Asymptomatic patients were not swabbed for COVID-19. For suspected or confirmed cases of COVID-19, minimum number of staff were allowed in the theatres. Patient interactions were kept to a minimum. During intubation, only the anesthetist and theatre ODP were allowed in the theatre. Between the operation and intubation, a 20 minutes buffer was taken to allow airflow clearance. This buffer was not required if full personal protection equipment was worn by all the staff members. No observers were allowed during these procedures. European urology stated that “decision for urgent or emergent surgeries should depend upon capacity and demand, but must also counterbalanced the effects of delaying surgery” (Stensland et al., 2020[12]).

### Outpatient clinic

All the clinics were converted to virtual clinics. Rescheduling or postponing of the clinic sessions were avoided where possible in order to prevent huge back log of patient in the post-COVID-19 periods. The clinics were run by consultants rather than registrars. This improved the decision-making process for the patients and reduced a lot of unnecessary follow-ups. On the other hand, those decisions were made based only on the patient's reported history as clinical examination and investigation could not be performed. The impact that this will have on diagnosis is still unknown.

## Discussion

COVID-19 is an invisible enemy which health care systems around the globe are trying to fight. While it continues to cause detrimental effects on every effect of life, both diagnosed and undiagnosed urological cancers could also be slowly killing people in the community. The expert guidelines mentioned above appear to be the safest way forward and we expect that by following these guidelines, we could tailor management that can balance both cancer intervention and diagnosis whilst limiting virus spread. At the moment, there is much to be learned about COVID-19.The current need at this time is to protect our community from COVID-19, prioritizing proper use of resources and keeping our health care system functioning.

## Conclusions

Overwhelming effects of COVID-19 on health care systems cannot be ignored. It has affected every aspect of health care in this society. We studied the effect of COVID-19 on the Department of Urology in one institute (Kings College Hospital, London). It affected both elective and emergency urology services. In order to limit the exposure of virus to patients, all elective clinics were either cancelled or converted to consultant led telephone consultations. Operating was limited to high grade cancer patients. The long-term impact of COVID-19 on urology is unknown. It is expected to increase the surgery waiting time. It is important to share the impact COVID-19 pandemic on urology services in order to prepare ourselves better and predict potential challenges in the future. There are many unknown effects at present of COVID-19 pandemic on the recovery of urological services, both elective and emergency. As we deviate from the current known protocols with regards to treatment time frame, the long-term outcomes of our patients treated during pandemic is yet to be known. With no known time-frame when normality can or will resume, we need to adapt our practices to provide patients with the safest urological care possible.

## Conflict of interest

The authors have no conflict of interest associated with this article.

## Funding

This study did not receive any external funding.

## References

[1] Babjuk M, Burger M, Compérat EM, Gontero P, Mostafid AH, Palou J (2019). European Association of Urology Guidelines on Non-muscle-invasive Bladder Cancer (TaT1 and Carcinoma In Situ) - 2019 update. Eur Urol.

[2] BAUS, British Association of Urological Surgeons (2020). COVID-19 bladder cancer contingency plan prepared by the BAUS Section of Oncology. Version 2: 31 March 2020. https://caunet.org/wp-content/uploads/2020/04/BAUS-Oncology-COVID-19-Bladder.pdf.

[3] BAUS, British Association of Urological Surgeons (2020). COVID-19 strategy for the interim management of kidney cancer. BAUS kidney cancer version 1. 2020. https://wmcanceralliance.nhs.uk/images/Documents/Covid-19_2020/COVID-19_BAUS_Oncology_Kidney_final.pdf.

[4] BAUS, British Association of Urological Surgeons (2020). COVID-19 strategy for the Interim management of prostate cancer prepared by the BAUS Section of Oncology. Version 1: 19 March 2020. cmcanceralliance.nhs.uk.

[5] Campi R, Amparore D, Capitanio U, Checcucci E, Salonia A, Fiori C (2020). Assessing the burden of nondeferrable major uro-oncologic surgery to guide prioritisation strategies during the COVID-19 pandemic: Insights from three italian high-volume referral centres. Eur Urol.

[6] Goossens-Laan CA, Leliveld AM, Verhoeven RHA, Kil PJM, De Bock GH, Hulshof MCCM (2014). Effects of age and comorbidity on treatment and survival of patients with muscle-invasive bladder cancer. Int J Cancer.

[7] Kamat AM, Bellmunt J, Galsky MD, Konety BR, Lamm DL, Langham D (2017). Society for Immunotherapy of Cancer consensus statement on immunotherapy for the treatment of bladder carcinoma. J Immunother Cancer. 2017;5(1):68. Erratum in: J Immunother Cancer.

[8] Klaassen Z, Kamat AM, Kassouf W, Gontero P, Villavicencio H, Bellmunt J (2018). Treatment Strategy for Newly Diagnosed T1 High-grade Bladder Urothelial Carcinoma: New Insights and Updated Recommendations. Eur Urol.

[9] Liang W, Guan W, Chen R, Wang W, Li J, Xu K (2020). Cancer patients in SARS-CoV-2 infection: a nationwide analysis in China. Lancet Oncol.

[10] Mano R, Vertosick EA, Hakimi AA, Sternberg IA, Sjoberg DD, Bernstein M (2016). The effect of delaying nephrectomy on oncologic outcomes in patients with renal tumors greater than 4 cm. Urol Oncol Semin Orig Investig.

[11] Miller KD, Nogueira L, Mariotto AB, Rowland JH, Yabroff KR, Alfano CM (2019). Cancer treatment and survivorship statistics, 2019. CA Cancer J Clin.

[12] Stensland KD, Morgan TM, Moinzadeh A, Lee CT, Briganti A, Catto JWF (2020). Considerations in the triage of urologic surgeries during the COVID-19 pandemic. Eur Urol.

[13] Thomas F, Noon AP, Rubin N, Goepel JR, Catto JWF (2013). Comparative outcomes of primary, recurrent, and progressive high-risk non-muscle-invasive bladder cancer. Eur Urol.

[14] Ward C (2020). COVID-19 Strategy for the Interim Management of Testicular Cancer Prepared by the BAUS Section of Oncology. Version 1: 22 March 2020.

